# Strain analysis is superior to wall thickening in discriminating between infarcted myocardium with and without microvascular obstruction

**DOI:** 10.1007/s00330-018-5493-0

**Published:** 2018-06-08

**Authors:** Henk Everaars, Lourens F. H. J. Robbers, Marco Götte, Pierre Croisille, Alexander Hirsch, Paul F. A. Teunissen, Peter M. van de Ven, Niels van Royen, Felix Zijlstra, Jan J. Piek, Albert C. van Rossum, Robin Nijveldt

**Affiliations:** 1 Department of Cardiology, VU University Medical Center, De Boelelaan 1117, 1081 HV Amsterdam, The Netherlands; 2 Netherlands Heart Institute (NHI), Utrecht, The Netherlands; 3 Creatis Medical Imaging Research Center, Lyon, France; 4 Department of Cardiology, Thoraxcenter, Erasmus Medical Center, Rotterdam, The Netherlands; 5 Department of Radiology, Erasmus Medical Center, Rotterdam, The Netherlands; 6 Department of Epidemiology and Biostatistics, VU University Medical Center, Amsterdam, The Netherlands; 7 Department of Cardiology, Amsterdam Medical Center, Amsterdam, The Netherlands

**Keywords:** ST elevation myocardial infarction, Magnetic resonance Imaging, Myocardial contraction, Left ventricular function

## Abstract

**Objectives:**

The aim of the present study was to evaluate the diagnostic performances of strain and wall thickening analysis in discriminating among three types of myocardium after acute myocardial infarction: non-infarcted myocardium, infarcted myocardium without microvascular obstruction (MVO) and infarcted myocardium with MVO.

**Methods:**

Seventy-one patients with a successfully treated ST-segment elevation myocardial infarction underwent cardiovascular magnetic resonance imaging at 2-6 days after reperfusion. The imaging protocol included conventional cine imaging, myocardial tissue tagging and late gadolinium enhancement. Regional circumferential and radial strain and associated strain rates were analyzed in a 16-segment model as were the absolute and relative wall thickening.

**Results:**

Hyperenhancement was detected in 418 (38%) of 1096 segments and was accompanied by MVO in 145 (35%) of hyperenhanced segments. Wall thickening, circumferential and radial strain were all significantly diminished in segments with hyperenhancement and decreased even further if MVO was also present (all *p* < 0.001). Peak circumferential strain (CS) surpassed all other strain and wall thickening parameters in its ability to discriminate between hyperenhanced and non-enhanced myocardium (all *p* < 0.05). Furthermore, CS was superior to both absolute and relative wall thickening in differentiating infarcted segments with MVO from infarcted segments without MVO (*p* = 0.02 and *p* = 0.001, respectively).

**Conclusions:**

Strain analysis is superior to wall thickening in differentiating between non-infarcted myocardium, infarcted myocardium without MVO and infarcted myocardium with MVO. Peak circumferential strain is the most accurate marker of regional function.

**Key Points:**

• *CMR can quantify regional myocardial function by analysis of wall thickening on cine images and strain analysis of tissue tagged images*.

• *Strain analysis is superior to wall thickening in differentiating between different degrees of myocardial injury after acute myocardial infarction*.

• *Peak circumferential strain is the most accurate marker of regional function*.

## Introduction

Although emergency percutaneous coronary intervention (PCI) in acute myocardial infarction (MI) restores epicardial coronary blood flow, microvascular perfusion is not restored in up to 40% of patients because of microvascular injury [1]. The presence of microvascular injury after primary PCI is associated with poor functional recovery and adverse events, with higher incidences of congestive heart failure and death [1–3]. Cardiovascular magnetic resonance imaging (CMR) allows accurate visualization of the region of microvascular injury, the so-called areas with microvascular obstruction (MVO) [4–7]. Among the range of available CMR techniques, late gadolinium enhancement (LGE) is the reference method for diagnosing MVO as it has been studied most extensively.

Although the detrimental effect of MVO on functional outcome is well established, there are limited data on the relationship between regional function and MVO [8]. Regional function can be assessed with CMR by analysis of wall thickening on steady-state free precession (SSFP) cine imaging [9]. Alternatively, regional function is quantified by analysis of strain using myocardial tissue tagging. In the pre-LGE era, myocardial strain was reported to be superior to wall thickening in differentiating dysfunctional from functional myocardium [10]. The accuracy of strain analysis in discriminating between different degrees of myocardial injury, as determined using LGE, is still unknown. Therefore, the aim of the present study was to compare the diagnostic performances of strain and wall thickening analysis in discriminating among three types of tissue: non-infarcted myocardium, infarcted myocardium without MVO and infarcted myocardium with MVO.

## Methods

### Patient population

This study is a pooled substudy of data from the PREDICT-MVO trial and from patients enrolled in the HEBE trial in the VU University Medical Center. The main results of these trials have been published before [11, 12]. In both studies, patients with a first ST-segment elevation myocardial infarction successfully treated by primary PCI within 12 h after onset of symptoms were included. The exclusion criteria were: (supra-)ventricular arrhythmias, cardiogenic shock, anticipated additional PCI or coronary artery bypass grafting within the next 4 months, unsuccessful PCI and known contraindications to CMR. Acute management and subsequent medical care followed contemporary guidelines [13]. Both trials were conducted in accordance with the Declaration of Helsinki, and protocols were approved by the Institutional Review Committee. All patients gave written informed consent for the study.

### CMR image acquisition

CMR was performed on a 1.5-T clinical scanner (Avanto, Siemens Healthineers) using a phased-array cardiac receiver coil. Imaging was scheduled between 3 to 6 days after reperfusion.

Cardiac function was assessed with cine imaging by using a segmented SSFP pulse sequence. Typical in-plane resolution was 1.3 × 1.3 mm^2^, with a slice thickness of 5 mm (repetition time 41-47 ms, echo time 1.6 ms, flip angle 60-75^°^, matrix 256 × 208 mm, temporal resolution < 40 ms).

Myocardial tissue tagging was performed using a multiple breath-hold retrospectively gated complementary spatial modulation of magnetization pulse (CSPAMM) sequence. The end-systolic phase of the horizontal long-axis cine series was used for planning tagged images in three parallel short-axis planes at the basal, mid and apical levels. Typical in-plane resolution was 1.2 × 1.2 mm^2^, with a 7-mm tag-tag distance and a slice thickness of 6 mm (repetition time 15-42 ms, echo time 1.2 ms, flip angle 20^°^, matrix 256 × 256 mm, temporal resolution 15-30 ms).

LGE was performed 12-15 min after administration of a gadolinium-based contrast agent (Dotarem®, Guerbet; 0.2 mmol/kg) using a two-dimensional segmented inversion recovery gradient-echo pulse sequence. The slice position was identical to the cine images. Typical in-plane resolution was 1.3 × 1.3 mm^2^, with a slice thickness of 5-6 mm (repetition time 8-9 ms, echo time 4.4 ms, flip angle 25^°^, matrix 256 × 200 mm, triggering every other heartbeat). The inversion time was set to null the signal of viable myocardium and typically ranged from 250-300 ms.

### CMR image analysis

Post-processing of CMR images was performed offline using dedicated software. Cine and LGE images were analyzed using Qmass (version 7.6, Medis Medical Imaging Systems). Tagged images were analyzed using inTag (Creatis Medical Imaging Research Center), which runs as a plug-in for OsiriX (version 6.5, Pixmeo) and is based on the sine-wave modeling (SinMod) technique [14]. Segmental data were analyzed using the 17-segment model of the American Heart Association [15], excluding segment 17 (apex).

On the short axis cine images, the endocardial and epicardial borders were manually outlined on the end-diastolic and end-systolic phase. From these, the end-diastolic volume (LVEDV), end-systolic volume (LVESV) and left ventricular ejection fraction (LVEF) were calculated. Segmental absolute and relative wall thickening (absWT and relWT) was computed by comparing end-diastolic and end-systolic wall thickness.

**Fig. 1:**
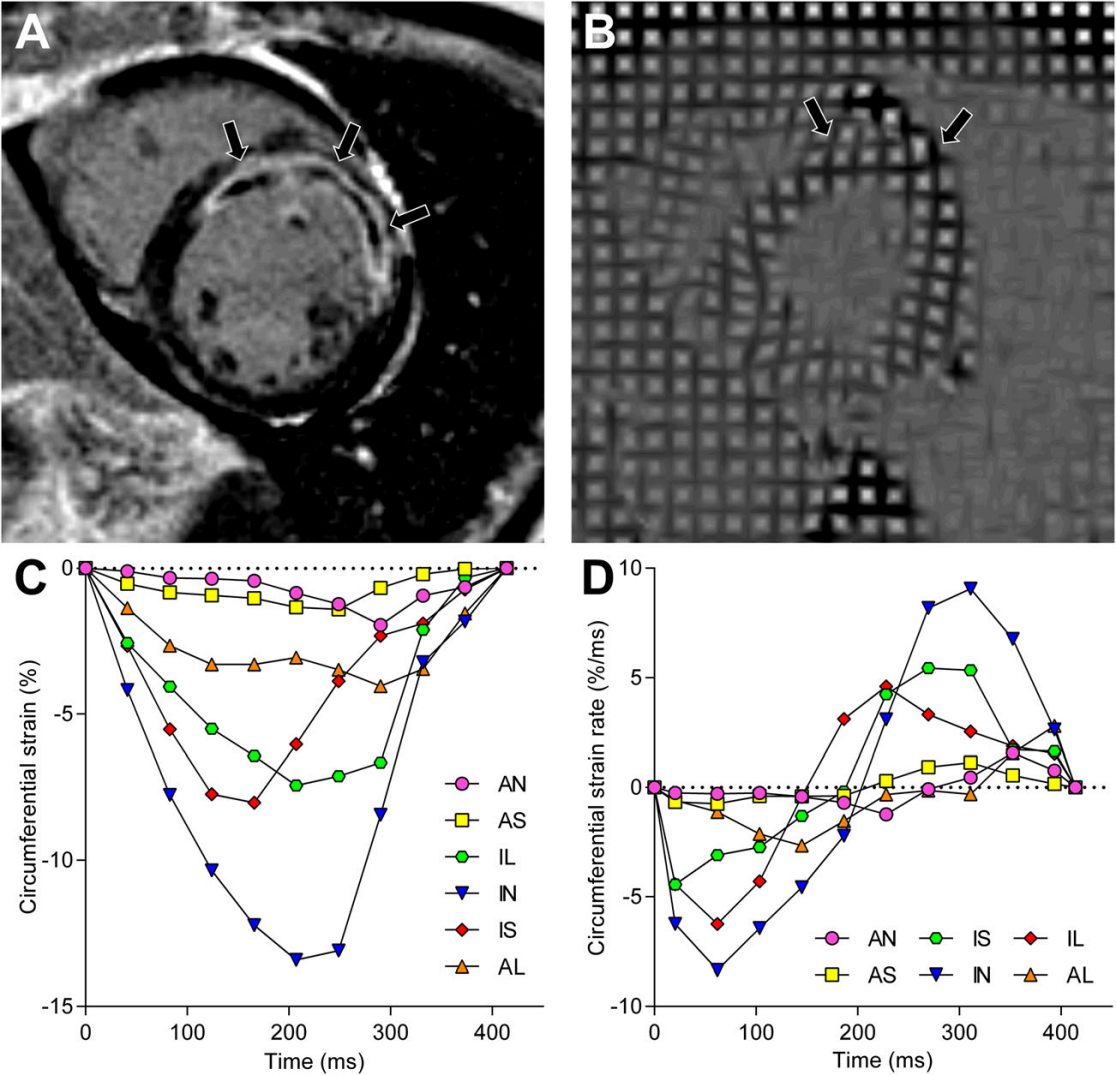
Example of strain and strain rate analysis in a patient after acute anterior myocardial infarction. Short-axis images (**A** and **B**) on identical locations 4 days after acute anterior myocardial infarction. The LGE image (**A**) demonstrates extensive hyperenhancement and MVO (arrows) of the anterior and anteroseptal wall. The end-systolic tagging image (**B**) shows limited deformation of the infarct zone (arrows), which is confirmed after strain analysis by a diminished peak circumferential strain (**C**) and reduced peak systolic and diastolic circumferential strain rates (**D**). AN = anterior, AS = anteroseptal, IS = inferoseptal, IN = inferior, IL = inferolateral, AL = anterolateral, MVO = microvascular obstruction

Strain analysis was performed by manually drawing the endocardial and epicardial borders on the end-systolic phase of the tagged images and propagating the contours to the other phases. Radial and circumferential strain were computed for every segment at each given phase of the cardiac cycle and plotted against time (Fig. 1A). Peak radial strain (RS) and peak circumferential strain (CS) were defined as the maximum and minimum of the radial and circumferential strain curves, respectively. Next, the slope of the strain curves was calculated at each phase and plotted against time (Fig. 1B). From these strain rate curves, the peak systolic and diastolic circumferential and radial strain rates (CSR and RSR, respectively) were determined.

Infarct size was calculated on the short-axis LGE images by using the full-width-at-half maximum method and is expressed as percentage of LV mass [16]. For every segment, the transmural extent of infarction was calculated by dividing the hyperenhanced area by the total area of the predefined segment. Segments were considered infarcted if ≥ 1% hyperenhancement was present. MVO was identified on the LGE images as a hypointense core within hyperenhanced myocardium. Areas of MVO were incorporated in the calculation of total infarct size. Remote myocardium was defined as the myocardial segments contralateral to the infarcted zone, without presence of hyperenhancement.

Analyses of cine, tagging and LGE images were performed independently from the results of the other techniques by a single observer (HE).

### Statistical analysis

Continuous variables are presented as mean ± standard deviation or median with interquartile range, whereas categorical variables are expressed as frequency with percentage. Log transformation was applied for total infarct size and transmural extent of infarction to achieve normal distribution for parametric testing. Pearson’s correlation was used to quantify the association between continuous variables. Means of continuous variables were compared between patients with and without MVO by using the Student *t* test. Means of segmental strain and wall thickening were compared between hyperenhanced and non-enhanced segments by means of a mixed linear model with a random effect for patient and slice nested within patient. A similar multilevel analysis was performed for comparing means of hyperenhanced segments with and without MVO and for comparing means of remote segments from patients with and without MVO. To correct for the transmural extent of infarction, an additional multilevel analysis was performed in which the transmural extent of infarction was added as a fixed factor. Furthermore, a mixed model analysis with the two-way interaction between MVO and transmural extent of infarction was performed to determine the effect of MVO on the relationship between CS and transmural extent of infarction. A receiver-operator characteristic (ROC) curve analysis and the Youden index were used to define cutoff values with the highest discriminative ability. Comparison of ROC curves was performed by using the method of deLong [17]. All statistical tests were two tailed, and *p* < 0.05 was considered statistically significant. Statistical analysis was done with SPSS (IBM SPSS Statistics 22 for Windows).

## Results

Myocardial tagging was performed in 50 of the 60 patients in the PREDICT-MVO trial and in all 23 patients of the HEBE trial enrolled in the VU University Medical Center. One patient was excluded from analysis because of contrast enhancement in a second coronary vascular territory and another patient was excluded because of poor image quality. Therefore, data of 71 patients were included in this analysis. In four patients, myocardial tissue tagging was only performed at the mid-level to reduce patient discomfort caused by repeated breath holding and therefore only 6 instead of 16 segments per patient were included.

Baseline characteristics of the patient cohort are listed in Table 1. Sixty-three percent of infarctions were related to the left anterior descending artery (LAD), 8% percent to the left circumflex artery (LCx) and 28% to the right coronary artery (RCA). MVO was present in 37 (52%) patients and was associated with higher peak CK-MB [208 U/l (112-324) vs. 120 U/l (33-209), *p* = 0.03]. Patients with MVO had larger LV ESV and LV EDV and lower LVEF (all *p* < 0.001). Additionally, infarct size was more than two-fold larger if MVO was present (25 ± 9 % vs. 11 ± 8 %; *p* < 0.001) and the amount of MVO correlated with infarct size (*r* = 0.65; *p* < 0.001).

**Table 1:** Baseline characteristics

Characteristic	*n* = 71
Age (years)	57 ± 10
Male gender	57 (80%)
Body mass index (kg/m^2^)	27 ± 3
Risk factors	
Family history of CAD	32 (45%)
Hypertension	17 (24%)
Hypercholesterolemia	9 (13%)
Diabetes mellitus	24 (34%)
Smoking	57 (80%)
Maximum CK-MB (U/l)	212 ± 224
Time to reperfusion (h)	2.4 [1.5-3.6]
TIMI flow grade 3 post PCI	67 (94%)
GP IIb/IIIa inhibitor used	24 (34%)
Infarct related artery	
LAD	45 (63%)
LCx	6 (8%)
RCA	20 (28%)
Medication at discharge	
ACE inhibitor or ATII antagonist	65 (92%)
Aspirin	71 (100%)
Beta-blocker	70 (99%)
P2Y12 inhibitor	71 (100%)
Statin	71 (100%)
LV volumes and LV function	
LV ED volume (ml/m^2^)^*^	93 ± 15
LV ES volume (ml/m^2^)^*^	49 ± 14
LV EF (%)	49 ± 8
Microvascular obstruction	37 (52%)

Data are mean ± standard deviation, absolute number (%) or median [interquartile range]. CAD = coronary artery disease; CK-MB = creatine kinase myocardial band; GP = glycoprotein; ACE = angiotensin-converting enzyme; ATII = angiotensin II receptor; LV ED = left ventricular end-diastolic; LV ES = left ventricular end-systolic; LV EF = left ventricular ejection fraction; * = indexed for body surface area

### Discriminating between infarcted and non-infarcted myocardium

A total of 1096 segments were analyzed of which 418 (38%) demonstrated hyperenhancement. As shown in Table 2, infarcted segments had reduced wall thickening and strain (all *p* < 0.001).

**Table 2:** Segmental function, stratified by presence of hyperenhancement

Characteristic	Hyperenhanced (*n* = 418)	Non-enhanced (*n* = 678)	*p* value
Wall thickening			
absWT (mm)	1.9 ± 1.7	4.0 ± 1.8	< 0.001
relWT (%)	27 ± 31	65 ± 41	< 0.001
Circumferential strain			
CS (%)	-6.6 ± 3.8	-12.7 ± 4.5	< 0.001
Systolic CSR (%/ms)	-4.6 ± 2.6	-7.6 ± 3.1	< 0.001
Diastolic CSR (%/ms)	5.0 ± 6.1	9.1 ± 4.6	< 0.001
Radial strain			
RS (%)	7.4 ± 6.5	15.6 ± 10.6	< 0.001
Systolic RSR (%/ms)	5.8 ± 5.0	9.4 ± 5.2	< 0.001
Diastolic RSR (%/ms)	-6.6 ± 4.0	-10.7 ± 6.0	< 0.001

Data as mean ± standard deviation. AbsWT= absolute wall thickening; relWT= relative wall thickening; CS = peak circumferential strain; CSR = circumferential strain rate; RS = peak radial strain; RSR = radial strain rate

**Fig. 2:**
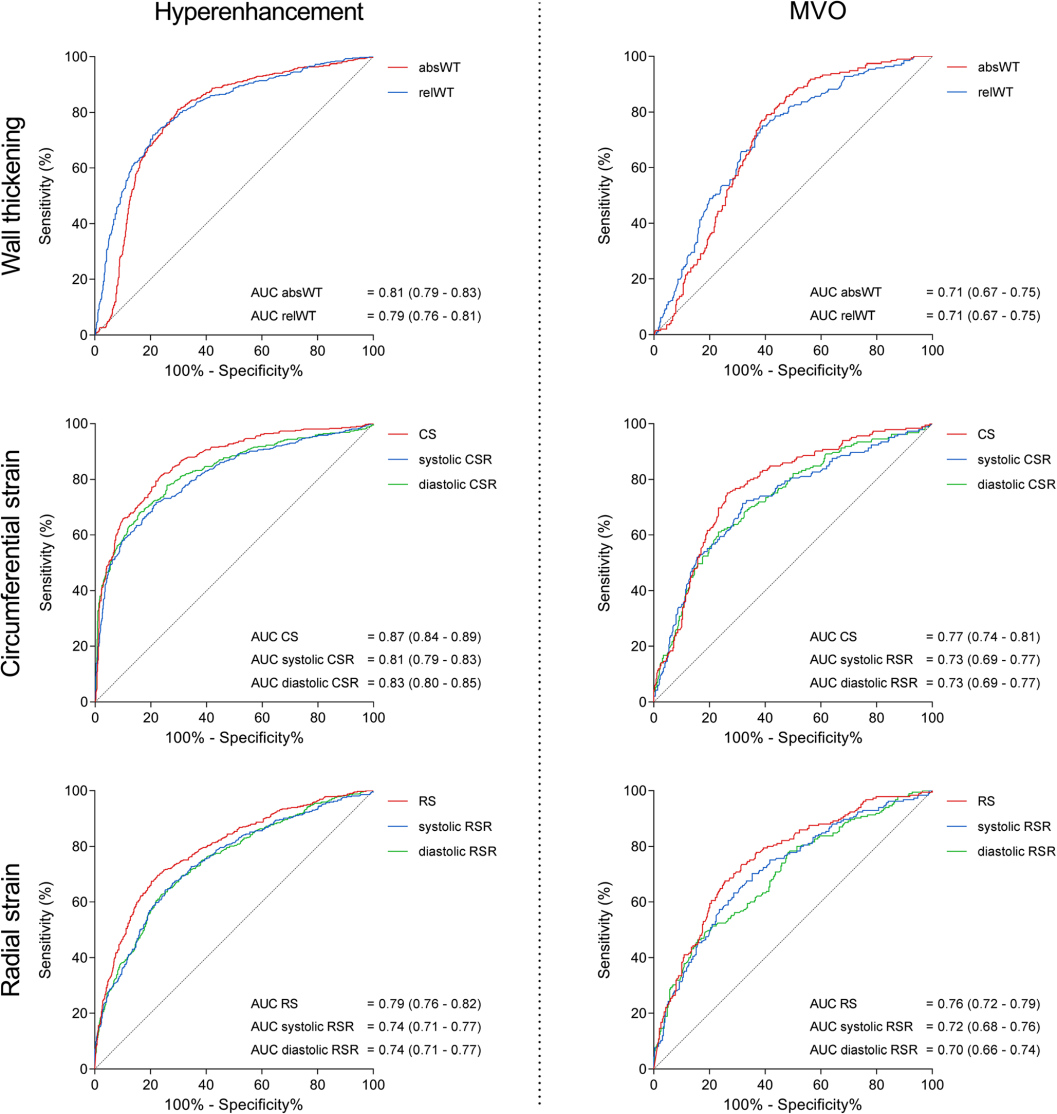
Receiver-operator characteristic curves of wall thickening and strain. Receiver-operator characteristic (ROC) curves with corresponding area under the curves (AUCs) and 95% confidence intervals of wall thickening (top row), circumferential strain (middle row) and radial strain (bottom row) for detecting hyperenhancement (left column) and microvascular obstruction (right column). AbsWT = absolute wall thickening; relWT = relative wall thickening; CS = peak circumferential strain; CSR = peak circumferential strain rate; RS = peak radial strain; RSR = peak radial strain rate

Figure 2 (left column) shows the ROC curves of wall thickening, circumferential and radial strain for discriminating between hyperenhanced and non-enhanced segments. Analysis of the ROC curves of wall thickening parameters revealed that the area under the curve (AUC) of absWT was significantly greater than the AUC of relWT (*p* = 0.004). CS had the highest test performance of the circumferential strain parameters, with a significantly greater AUC than both systolic CSR (*p* < 0.001) and diastolic CSR (*p* = 0.001). Within the group of radial strain parameters, RS outperformed systolic and diastolic RSR (both *p* < 0.001).

To define the optimal parameter for discriminating between non-infarcted and infarcted myocardium, we selected the most accurate wall thickening, radial strain and circumferential strain parameters and compared their diagnostic performances. CS surpassed RS and absWT in its ability to discriminate hyperenhanced from non-enhanced myocardium (both *p* < 0.001). No difference was observed between the test performance of RS and absWT (*p* = 0.12). Regarding diastolic function, CSR outperformed RSR (*p* < 0.001) in discriminating between infarcted and non-infarcted myocardium.

### Discriminating between infarcted myocardium with and without MVO

Of the 418 segments with hyperenhancement, 145 (35%) demonstrated MVO. As shown in Table 3, infarcted segments with MVO had impaired strain and wall thickening compared with infarcted segments without MVO (all *p* < 0.001). However, transmural extent of infarction was significantly higher in segments with MVO [65% (42-85) vs. 14% (5-35), *p* < 0.001]. After correcting for transmural extent of infarction, segments with MVO were still found to have reduced circumferential strain parameters and radial strain rates compared with segments without MVO (Table 3). In contrast, wall thickening parameters and RS no longer differed between segments with and without MVO (all *p* > 0.05).

**Table 3:** Deformation of infarcted segments, stratified by presence of MVO

Characteristic	MVO (*n* = 145)	No MVO (*n* = 273)	Uncorrected *p* value	Corrected *p* value
Wall thickening				
absWT (mm)	1.4 ± 1.4	2.3 ± 1.7	< 0.001	0.657
relWT (%)	17 ± 22	34 ± 34	< 0.001	0.148
Circumferential strain				
CS (%)	-5.3 ± 3.4	-7.6 ± 3.7	< 0.001	<0.001
Systolic CSR (%/ms)	-4.0 ± 2.7	-5.1 ± 2.4	< 0.001	<0.001
Diastolic CSR (%/ms)	4.0 ± 4.2	5.8 ± 7.1	< 0.001	0.019
Radial strain				
RS (%)	4.9 ± 4.6	9.3 ± 7.0	< 0.001	0.152
Systolic RSR (%/ms)	4.6 ± 4.0	6.8 ± 5.5	< 0.001	0.011
Diastolic RSR (%/ms)	-5.4 ± 3.5	-7.5 ± 4.2	< 0.001	0.024
Transmural extent of infarction (%)	64.9 [42.3-84.7]	13.9 [4.7-34.8]	< 0.001	

Data as mean ± standard deviation or median [interquartile range]. The far right column displays the *p* values after correction for transmural extent of infarction. Abbreviations as in Table 2

Figure 2 (right column) shows the ROC curves of wall thickening, circumferential and radial strain for differentiating between hyperenhanced segments with and without MVO. The test performance of absWT was similar to relWT (*p* = 0.74). For circumferential strain, CS had greater diagnostic potential than systolic CSR (*p* = 0.004) and diastolic CSR (*p* = 0.02). RS was the most accurate radial strain parameter, outperforming systolic RSR (*p* = 0.02) as well as diastolic RSR (*p* = 0.01).

To reveal the parameter with the highest discriminative power, we compared the diagnostic performances of CS, RS and absWT. CS had a significantly greater AUC than both absWT (*p* = 0.02) and relWT (*p* = 0.01), but was comparable to RS (*p* = 0.43) in its ability to differentiate between hyperenhanced segments with and without MVO.

**Fig. 3:**
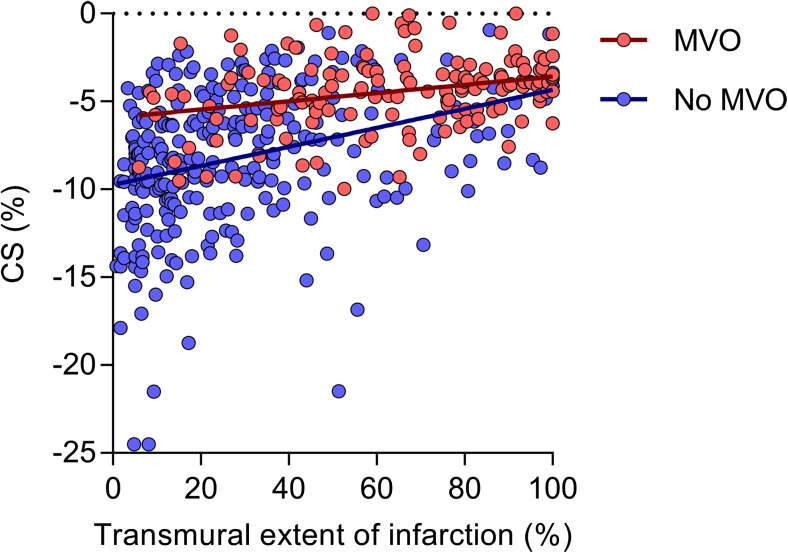
Relationship between CS and transmural extent of infarction. Scatterplot demonstrating the relationship between CS and transmural extent of infarction in hyperenhanced segments with (**red**) and without MVO (**blue**). Abbreviations as in Figs. 1 and 2

Figure 3 displays the relationship between CS and transmural extent of infarction in hyperenhanced segments with and without MVO. Presence of MVO was a significant effect modifier in the relation between CS and transmural extent of infarction (*p* = 0.009). In segments with MVO the fitted linear model was CS = -6.11 + 0.02 × transmural extent of infarction (*p*-value for the regression coefficient of transmural extent of infarction < 0.001), whereas in segments without MVO: CS = -9.81 + 0.06 × transmural extent of infarction (*p* < 0.001).

### Optimal cutoff values and diagnostic accuracies

Table 4 lists the optimal cutoff values of strain and wall thickening parameters for differentiating among the three types of tissue. The sensitivity, specificity, positive predictive value, negative predictive value and accuracy achieved when using these cutoff values are also shown. CS had an optimal cutoff value of -6.2% for differentiating between hyperenhanced segments with and without MVO. Using this cutoff, CS achieved an accuracy of 78%. Wall thickening analysis on the other hand had modest accuracies of 65% (absWT) and 66% (relWT) for discriminating between infarcted segments with and without MVO.

**Table 4:** Optimal cut-off values and diagnostic performance of strain and wall thickening for detecting hyperenhancement and detecting microvascular injury within hyperenhanced segments

Characteristic	Hyperenhancement						MVO					
	Optimal cut-off	Sensitivity (%)	Specificity (%)	PPV (%)	NPV (%)	Accuracy (%)	Optimal cut-off	Sensitivity (%)	Specificity (%)	PPV (%)	NPV (%)	Accuracy (%)
*Wall thickening*												
absWT	2.7 mm	72	79	68	82	76	2.1 mm	75	61	51	82	65
relWT	48 %	81	70	63	86	74	31 %	79	60	51	84	66
*Circumferential strain*												
CS	-9.6 %	81	76	68	87	78	-6.2 %	75	74	60	85	74
Systolic CSR	-5.9 %/ms	72	78	67	82	76	-4.7 %/ms	71	68	54	82	69
Diastolic CSR	5.5 %/ms	68	84	72	81	77	3.5 %/ms	61	77	58	79	71
*Radial strain*												
RS	8.1 %	68	80	67	80	75	5.7 %	68	74	58	81	72
Systolic RSR	5.8 %/ms	64	74	61	77	70	5.0 %/ms	70	65	51	80	66
Diastolic RSR	-6.8 %/ms	63	76	62	77	71	-4.7 %/ms	50	82	59	75	70

PPV = positive predictive value; NPV = negative predictive value; other abbreviations as in Table 2 and 3

### Strain in remote myocardium

Strain and wall thickening in remote segments did not differ between patients with and without MVO (all *p* > 0.05; -12.8 ± 4% vs. -12.6 ± 4% for CS). In addition, strain and wall thickening of remote segments were not associated with infarct size (all *p* > 0.05; CS = - 14.61 + 0.04 × infarct size).

## Discussion

Our study is the largest to date in which myocardial tissue tagging was performed in patients after successful primary stenting for acute MI. We compared the diagnostic performances of multiple strain and wall thickening parameters in differentiating among three types of tissue: non-infarcted myocardium, infarcted myocardium without MVO and infarcted myocardium with MVO. Our main findings can be summarized as follows:

(1) Circumferential strain is superior to radial strain and wall thickening in discriminating infarcted from non-infarcted myocardium as well as in discriminating between infarcted myocardium with and without MVO. (2) CS is superior to systolic and diastolic CSR and consequently is the most accurate tool for quantifying regional function. (3) After correcting for transmural extent of infarction, circumferential strain parameters and peak radial strain rates are still reduced in segments with MVO. (4) Strain in remote myocardium does not differ between patients with and without MVO and is not associated with infarct size.

### Wall thickening

Functional loss of myocardium is the hallmark of acute MI [18, 19], and although global measures yield important prognostic information, they have low sensitivity for detecting regional dysfunction [20, 21]. Analysis of wall thickening on cine images has traditionally been used as the reference technique for quantifying regional function [9, 22, 23]. This technique, however, has several limitations. The conical shape of the LV causes the short-axis images and myocardial wall to intersect at an oblique angle, leading to overestimation of wall thickness [24]. In addition, folding of trabeculae during systole makes it difficult to identify the end-systolic endocardial border and, as a consequence, wall thickening analysis is subject to high interobserver variability [25]. Myocardial tissue tagging overcomes these limitations by allowing direct tracking of intramural deformation after application of a saturation grid [26, 27]. Götte et al. were the first to compare the diagnostic capabilities of strain and wall thickening in patients after reperfused MI [10]. As contrast-enhanced CMR was not performed, segments were classified as pertaining to the infarct-related, adjacent or remote area according to a pre-specified distribution of coronary perfusion. Wall thickening in the infarct-related area was not significantly lower than wall thickening in the remote area, whereas both circumferential and radial strains were severely impaired. In the present study, the infarct-related area was defined as all segments containing hyperenhancement. We did find a significant decrease of wall thickening in segments with hyperenhancement. Strain analysis outperformed wall thickening nonetheless. We also found that circumferential strain was superior to radial strain in differentiating between different degrees of myocardial injury. This is not surprising as contraction of the myocardial fibers is predominantly oriented in the circumferential direction [28, 29]. In addition, the distance between the endo- and epicardium is relatively small compared with the circumferential diameter, resulting in fewer intersection points of the tagging grid in the radial dimension. This is even more problematic in the chronic phase of infarction, as post-infarct LV remodeling results in thinning of transmurally infarcted myocardium. Radial strain is therefore more sensitive to noise, and this also explains the higher standard deviation of radial strain parameters reported in previous studies and observed in the present study [10, 30]. On the other hand, myocardial tethering of the peri-infarct zone affects circumferential strain more than radial strain.

### Detrimental effect of MVO

Previous studies investigating the effect of MVO on myocardial function have reported that microvascular injury is independently associated with poor prognosis and reduced global function [2, 3, 31, 32]. Limited data exist on the relationship between MVO and regional function. Nijveldt et al. demonstrated that transmurally infarcted segments with MVO are less likely to recover at 3-month follow-up than transmurally infarcted segments without MVO [8]. Kidambi et al. obtained serial CMR scans in 39 STEMI patients and observed that from day 7 onwards MVO was independent from infarct size associated with attenuated strain in the infarct zone [33]. Finally, Bergerot et al. performed contrast-enhanced CMR and echocardiography speckle tracking in the acute phase of infarction. Segmental longitudinal strain was reduced in infarcted segments with MVO, independent from the transmural extent of infarction [34]. The findings of the present study also indicate that MVO reduces contractile function in the early phase after reperfusion. At 3-6 days after infarction, strain parameters were reduced in segments with MVO, even after correcting for transmural extent of infarction. Furthermore, MVO was a significant effect modifier in the relationship between CS and transmural extent of infarction. In contrast, strain in remote myocardium was unaltered in the presence of MVO, a finding that is also in line with previous observations [33]. MVO confers its detrimental effect directly to the infarcted myocardium, and this process starts within days after reperfusion. However, the pathophysiological mechanisms by which MVO affects contractile function have yet to be elucidated.

### Strain analysis

Strain analysis using myocardial tissue tagging is the current gold standard for quantifying regional myocardial function. The disadvantage of tissue tagging however is that dedicated sequences are needed, which prolong scanning duration and are not available at every imaging center. Additionally, laborious post-processing is required. Myocardial tagging acquisition has therefore primarily been confined to the research environment. Myocardial feature tracking is a promising novel technique that allows for analysis of myocardial strain using standard cine images, which are routinely part of almost every CMR examination [35]. Thus far, studies comparing feature tracking and tissue tagging strain have yielded conflicting results, ranging from very poor to excellent agreement [36–38]. In these studies however, feature tracking was performed using cine images acquired at relatively low spatial and temporal resolution. At these low resolutions, CMR is not able to differentiate features within the myocardial wall and feature tracking may not be performed reliably. New studies are therefore warranted, in which tissue tagging and feature tracking strain, derived from high temporal resolution cine images, are compared head to head in relation to findings on LGE images. In these studies, our reported diagnostic accuracies and cutoff values could serve as a guide. Finally, automated post-processing software has to be developed before strain analysis can be adopted into the clinic.

### Study limitations

Our study has several limitations. Strain was calculated for the entire radial dimension of the LV wall. In acute MI, irreversible myocyte damage develops in the subendocardium and progresses toward the subepicardium with increasing duration of coronary occlusion, the so-called wavefront phenomenon [39]. We may have underestimated the full diagnostic potential of strain parameters by not stratifying strain according to myocardial layer. Second, our defined cutoff values are based on the presence of hyperenhancement on LGE images, even though severe contractile dysfunction after MI can also occur in the absence of myocyte necrosis, a phenomenon known as myocardial stunning [40]. Conversely, contractile function may be preserved in myocardium with limited hyperenhancement. Furthermore, strain parameters describe myocardial deformation, which is determined by both active contraction of myocardial fibers and passive movement. Myocardial deformation is therefore dependent on loading conditions and does not equate to intrinsic contractile function. In addition, strain analysis is susceptible to significant user dependency. In normal hearts, both radial and circumferential strain increase tremendously from the epicardium to the endocardium, making accurate and reproducible delineation of contours extremely important [30].

## Conclusions

Strain analysis is superior to wall thickening in discriminating between infarcted and non-infarcted myocardium and in discriminating infarcted myocardium with MVO from infarcted myocardium without MVO. Peak circumferential strain is the most accurate marker of regional function.

## Abbreviations

AbsWT: Absolute wall thickening
AUC: Area under the curve
CMR: Cardiovascular magnetic resonance imaging
CS: Peak circumferential strain
CSPAMM: Complementary spatial modulation of magnetization
CSR: Peak circumferential strain rate
LAD: Left anterior descending artery
LGE: Late gadolinium enhancement
LV: Left ventricle
LVEF: Left ventricular ejection fraction
LVEDV: Left ventricular end-diastolic volume
LVESV: Left ventricular end-systolic volume
MI: Myocardial infarction
MVO: Microvascular obstruction
PCI: Percutaneous coronary intervention
RelWT: Relative wall thickening
RCA: Right coronary artery
ROC: Receiver-operating characteristic
RS: Peak radial strain
RSR: Peak radial strain rate
SinMod: Sine-wave modeling
SSFP: Steady-state free precession
STEMI: ST-segment elevation myocardial infarction
